# Difference in Leukocyte Composition between Women before and after Menopausal Age, and Distinct Sexual Dimorphism

**DOI:** 10.1371/journal.pone.0162953

**Published:** 2016-09-22

**Authors:** Yequn Chen, Yanhong Zhang, Guojun Zhao, Chang Chen, Peixuan Yang, Shu Ye, Xuerui Tan

**Affiliations:** 1 First Affiliated Hospital of Shantou University Medical College, Shantou, China; 2 Shantou University Medical College, Shantou, China; 3 Department of Cardiovascular Sciences, University of Leicester, Leicester, United Kingdom; 4 NIHR Biomedical Research Centre in Cardiovascular Disease, Leicester, United Kingdom; Stellenbosch University Faculty of Medicine and Health Sciences, SOUTH AFRICA

## Abstract

There are sex differences in many inflammatory and immune diseases, and the differences tend to diminish after menopause. The underlying reasons are unclear, but sex hormone levels are likely to be an important factor. Blood leukocyte count and composition provide an indicator of the inflammatory and immune status of an individual. We performed a cross-sectional analysis of blood leukocyte data from 46,879 individuals (26,212 men and 20,667 women, aged 18 to 93 years) who underwent a routine health checkup. In women aged around 50 years, neutrophil percentage (NE%) dropped whilst lymphocyte percentage (LY%) rose. Accordingly, women before age 50 had significantly higher NE%, lower LY%, and higher neutrophil-to-lymphocyte ratio (NLR) than women of 51–70 years of age (*p* = 1.35×10^−82^, *p* = 5.32×10^−100^, and *p* = 1.25×10^−26^, respectively). In age groups of <50 years, women had higher NE%, lower LY% and higher NLR than men (*p* = 1.82×10^−206^, *p* = 1.46×10^−69^, and *p* = 2.30×10^−118^, respectively), whereas in age groups of >51 years, it was the reverse (*p* = 1.92×10^−15^, *p* = 1.43×10^−84^, and *p* = 1.51×10^−48^, respectively). These results show that blood leukocyte composition differs between women before and after menopausal age, with distinct sexual dimorphism.

## Introduction

It has been well documented that there are significant differences between men and women in the incidence, onset of symptoms and severity of a number of diseases. In particular, men have higher incidence of coronary heart disease (CHD)[1–3] and abdominal aortic aneurysms [4], whereas women have higher rates of several autoimmune disorders including systemic lupus erythematosus, multiple sclerosis, and rheumatoid arthritis [5, 6]. Interestingly, although men have higher CHD incidence, female patients with myocardial infarction have greater infarction recurrence rates and mortality than male cases [7–11]. In multiple sclerosis, women have earlier onset of symptoms than men, but men tend to exhibit a more progressive and severe disease course [5].

The biological mechanism underlying sexual dimorphism in disease incidence and outcome is incompletely understood. Many of the diseases with sex differences, such as those mentioned above, are caused by inflammation and altered immunity. Studies have shown that males and females differ in their responses to inflammatory stimuli [5, 6, 12, 13]. There is substantial evidence indicating that this is, to a large extent, attributed to the effects of sex hormones, especially, estrogens [5, 6, 12, 13].

Leukocytes play various roles in inflammation and immunity. Neutrophils and monocytes are key players in innate immune system, with neutrophils being the first-responders of inflammatory cells to migrate towards the site of inflammation at the acute phase. Lymphocytes play central roles in adaptive immune system, with T lymphocytes being instrumental to cell-mediated immune response whilst B lymphocytes being responsible for production of antibodies in humoral immunity. Eosinophils and basophils are involved in allergy.

Blood leukocyte count and composition provide an important indicator of the inflammatory and immune status of an individual. Abnormalities in the numbers of certain types of leukocyte have been noted in studies of patients with some sexually dimorphic diseases. For example, studies have shown that patients with CHD have increased blood neutrophils and monocytes and decreased lymphocytes [14–18] and that the neutrophil-to-lymphocyte ratio (NLR) is a good predictor of cardiovascular risk and outcomes [16, 17, 19–27].

Intriguingly, studies have shown that the differences between men and women in incidence and clinical presentation of a number of diseases tend to diminish after the menopausal age. For instance, the higher mortality of myocardial infarction in female patients than in male cases is mainly observed before the menopausal age (<50–55 years of age) [7–11]. Similarly, the high female to male incidence rate for systemic lupus erythematosus is dramatically reduced following menopause [28].

The reason for age-dependent sexual dimorphism is unknown. Since inflammation and altered immunity plays important roles in many diseases with sex bias, we sought to investigate whether there were age-dependent differences between men and women in the numbers and percentages of different types of leukocyte in the blood. To address this question, we studied a large cohort of individuals and ascertained differences among age groups in men and women, respectively, and differences between men and women in various age groups.

## Methods

### Subjects

We studied hospital records of 46,879 healthy individuals (high school and university students, and current and retired employees of the public and private sectors) undergoing a routine health checkup at the First Affiliated Hospital of Shantou University Medical College, from 2011 to 2014. We collected demographic data including age, sex, total leukocyte counts, counts and percentages of monocytes, neutrophils, eosinophils, basophils, and lymphocytes. Hematological measurements were conducted by the hospital clinical chemistry department. All subjects in this study were Chinese. The study was approved by the Ethics Committee of the First Affiliated Hospital of Shantou University Medical College. This was a retrospective analysis of records. Although written content was not sought, records/information of the study subjects were anonymized prior to analysis.

### Statistical analysis

NLR was calculated by dividing neutrophil count by lymphocyte count. Values of total leukocyte counts, monocyte counts and percentages, neutrophils counts, eosinophil counts and percentages, basophil counts and percentage, lymphocyte counts, and NLR, were logarithmically transformed to normalize their distributions. T-tests were performed to ascertain differences among age groups and between men and women in total leukocyte count, counts and percentages of different leukocyte types, and NLR. All *p*-values were two sided.

## Results

The study included 46,879 subjects of between 18 and 93 years of age [26,212 men, mean age 42.60 years (standard deviation 15.05); and 20,667 women, mean age 41.76 (standard deviation 14.84)]. The subjects were divided into different age groups in men and women separately, in analyses to ascertain: 1) differences among age groups in men and women, respectively, and 2) differences between men and women in different age groups, in total leukocyte count, counts and percentages of leukocyte types, and NLR.

### Differences among age groups

**Fig 1:**
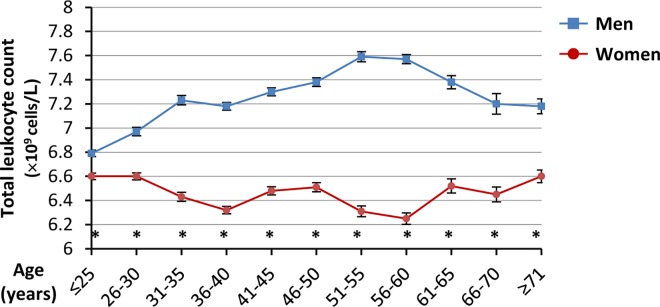
Total leukocyte counts in men and women in different age groups. Data shown are mean ± standard error of mean in men and women, respectively, in each age group; *indicates p<0.05 comparing men and women in the respective age group.

**Fig 2:**
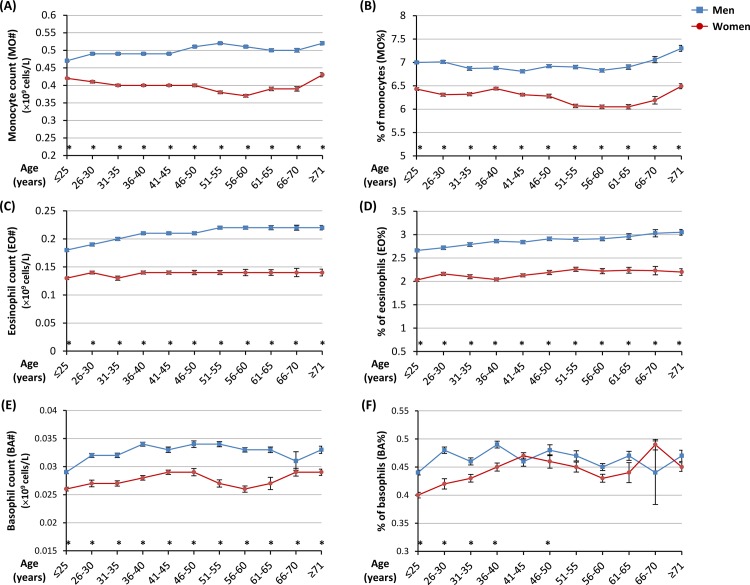
Counts and percentages of monocyte, eosinophil and basophil in men and women in different age groups. Data shown are mean ± standard error of mean in men and women, respectively, in each age group; *indicates p<0.05 comparing men and women in the respective age group.

In men, total leukocyte counts increased with increasing age up to age 55 and then gradually decreased, whereas in women, total leukocyte counts appeared to have a bimodal distribution (Fig 1 and S1 Table). Monocyte counts and percentages (MO# and MO%), eosinophil counts and percentages (EO# and EO%), and basophil counts and percentages (BA# and BA%) were similar among different age groups in both men and women (Fig 2 and S2–S4 Tables).

**Fig 3:**
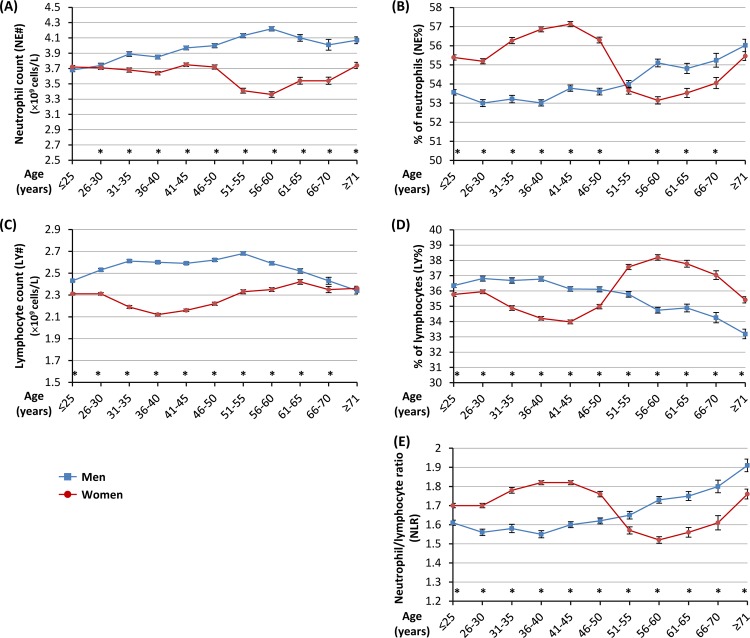
Counts and percentages of neutrophil and lymphocyte, and neutrophil-to-lymphocyte ratio, in men and women in different age groups. Data shown are mean ± standard error of mean in men and women, respectively, in each age group; *indicates p<0.05 comparing men and women in the respective age group.

In men, neutrophil counts and percentages (NE# and NE%) tended to increase with increasing age, whilst lymphocyte counts and percentages (LY# and LY%) decreased after age 51 (Fig 3 and S5 and S6 Tables). Accordingly, NLR rose with increasing age (Fig 3 and S7 Table).

In women of around 50 years of age, there was a significant drop of NE% and a substantial rise of LY%, with a considerable fall in NLR (Fig 3 and S5–S7 Tables). Accordingly, overall women of <50 years of age had significantly higher NE%, lower LY% and higher NLR, than women of 51–70 years of age (*p* = 1.35×10^−82^, *p* = 5.32×10^−100^, and *p* = 1.25×10^−26^, respectively). However, after age 56, NE% gradually increased while LY% declined, with a steady rise of NLR (Fig 3 and S5–S7 Tables).

### Differences between men and women

In almost all age groups studied, men had higher total leukocyte counts, MO#, MO%, NE#, LY#, EO#, EO%, and BA# (Figs 1 and 2 and S1–S4 Tables).

However, there was a significant age-dependent sexual dimorphism in NE%, LY% and NLR (*p* = 2.24×10^−60^, *p* = 2.51×10^−82^, and *p* = 1.11×10^−74^, respectively, for tests of interactions between age and sex). In the age groups of <50 years, woman had higher NE%, lower LY% and higher NLR, than man (*p* = 1.82×10^−206^, *p* = 1.46×10^−69^, and *p* = 2.30×10^−118^, respectively), whereas in the age groups of >51 years, the differences were in the reverse direction, i.e. women had lower NE%, higher LY% and lower NLR, than men (*p* = 1.92×10^−15^, *p* = 1.43×10^−84^, and *p* = 1.51×10^−48^, respectively) (Fig 3 and S5–S7 Tables).

## Discussion

There are several novel findings from this study: 1) in women aged around 50, NE% drops whilst LY% rises, with a considerable fall in NLR; 2) women before age 50 have significantly higher NE%, lower LY%, and higher NLR than women of 51–70 years of age; 3) in age groups of <50 years, women have higher NE%, lower LY% and higher NLR than men, whereas in age groups of >51 years, it is the reverse; and 4) in men after age 51 and in women after age 56, NE% gradually increases while LY% continuously declines, with a steady rise of NLR.

The abovementioned changes and differences in NE% and LY% in various age groups with distinct patterns in the two sexes are likely to arise from several reasons. First, studies have shown that in women, the estradiol level dramatically falls (by ~70%) during menopause which commonly occurs around 50 years of age [12, 29]. Estradiol has been shown to delay neutrophil apoptosis [30] and reduce lymphocyte production in the bone marrow [31, 32]. Therefore, after menopause, the significant reduction of estradiol level will likely result in higher neutrophil apoptotic rates and increased lymphocyte production, leading to reduced NE% and risen LY%. This can explain the aforementioned findings of our study that there is a significant drop of NE% and substantial rise of LY% in women of around 50 years of age and that women of <50 years of age have significantly higher NE% and lower LY% than women of 51–70 years of age.

Second, women before menopause have higher estrogen levels than men, however, men can have higher levels of estrogen than postmenopausal women due to the age-associated increased aromatization of testosterone in men and significantly reduced estrogen production in postmenopausal women [12]. This provides a possible explanation for the finding of our study that before age 50, women have higher NE% and lower LY% than men, whereas in age groups of 51–70 years, it is the reverse.

Third, studies have shown that the ageing process is associated with changes in the composition and function of the immune system, resulting in elevated levels of basal inflammation and impaired ability to mount efficient innate and adaptive immune responses to pathogens [12, 33]. Although the numbers of immune cells appear to remain stable with age [33, 34], haematopoietic stem cells in aged mouse are biased towards myeloid differentiation at the expense of lymphopoiesis, and there is also evidence for a similar skewing in elderly humans [33, 35]. This can explain the finding of our study that in men after age 51 and in women after 56, NE% gradually increases while LY% continuously declines, with a steady rise of NLR. Furthermore, the observation in our study that in the age groups of >51 years, men had higher NE% and lower LY% than women is in line with the notion proposed by other researchers that overall, males experience more prominent ageing related changes in the immune system that females, for example, with more pronounced reduction in lymphocytes [12].

Several previous studies of blood leukocytes had reported sex differences in the levels of some types of leukocyte. In a study of European men (n = 100, median ages of 24 years) and women (n = 100, median ages of 25 years), blood neutrophil counts were found to be higher in women [36]. Similarly, higher neutrophil counts in females were observed in a study of Africans (216 men and 201 women) of 18–55 years of age [37]. In a study of healthy white (n = 663), black (n = 697), Latin-American (n = 535) and Asian (n = 247) adults, women had higher granulocyte counts but lower monocyte counts [38]. However, these previous reports did not provide data stratified into different age groups. In present study, we analyzed data from a large group of individuals with a wide age range (26,482 Chinese men and 20,872 Chinese women, age from 18 to 93). The substantially larger sample size of our study provided greater statistical power and precision. Furthermore, the large sample size and broad age range of our study subjects allowed us to test sex differences in different age groups. This enabled us to uncover the age-dependent sexual dimorphism in NE%, LY% and NLR.

The finding of the present study that there is age-dependent sexual dimorphism in blood leukocyte composition points to a need to consider age as an important factor in future research that investigates into sex differences in immune system and the pathogenesis of inflammatory and immune diseases. Women generally have lower incidence of infections but higher rates of autoimmune diseases than men, but the differences diminish after the menopausal age [5, 6, 12, 13]. Previous studies have indicated that women generally have more vigorous immune and inflammatory responses than men [5, 6, 12, 13]. However, it is unclear if this is the case across all age groups. Further investigations to address this would be warranted. Moreover, further studies are required to investigate whether sex bias in leukocyte functions and inflammatory molecule productions is consistent among all age groups or has different patterns in different age groups.

The findings of our study may help explain some of the sexually dimorphic clinical situations. For example, studies have shown that before age 50–55, female patients with myocardial infarction have higher recurrent infarction rates and mortality than male cases [7–11], the reason for which is unknown. The finding of our study that women before age 50 have higher NLR than men in the same age groups provides an explanation. Since high NLR is a risk factor and an independent predictor for increased future cardiovascular events and mortality in CHD patients as demonstrated in many studies [16, 17, 19–27], it is plausible that the higher recurrent infarction rates and mortality in female myocardial infarction cases before age 50 are related to elevated NLR, as compared with male cases of the same age.

The finding that there is a dramatic drop of NE% and NLR and a significant raise of LY% in women after the menopausal age could also help explain the adverse effect of hormone replacement therapy in postmenopausal women. Studies have shown that combined estrogen and progestogen therapy in postmenopausal women increases the rates of CHD [39, 40]. This could be related to an increase of NLR (a risk factor for CHD) caused by the estrogen and progestogen intake in the postmenopausal women who would otherwise have low NLR. Although not specifically designed for investigation in this context, a previous study showed that administration of progesterone increased the numbers of circulating neutrophils while decreasing lymphocyte proportions in the blood [41] and another study showed that combined estrogen and progestogen therapy caused reduced blood lymphocyte counts in postmenopausal women [42].

However, it is noteworthy that the mean differences in NE% and LY% between males and females in the different age groups observed in this population cross-sectional study were only moderate, being <10%. Whether or not these moderate differences in NE%, LY% and NLR contribute to the sex differences in clinical manifestations mentioned above in individual patients is unclear. The dataset available in our study cannot address this question, and further studies to investigate this question would be warranted.

In summary, our study reveals that blood leukocyte composition differs between women before and after menopausal age, with distinct sexual dimorphism. These findings suggest a need to consider age as an important factor in future studies of sex differences in inflammation and immunity and related diseases.

## Supporting Information

S1 Table Total leukocyte counts in men and women in different age groups. (DOCX)

S2 Table Monocyte counts and percentages in men and women in different age groups. (DOCX)

S3 Table Eosinophil counts and percentages in men and women in different age groups. (DOCX)

S4 Table Basophil Counts and percentages counts in men and women in different age groups. (DOCX)

S5 Table Neutrophil counts and percentages in men and women in different age groups. (DOCX)

S6 Table Lymphocyte counts and percentages in men and women in different age groups. (DOCX)

S7 Table Neutrophil-to-lymphocyte ratio in men and women in different age groups. (DOCX)
