# Supplementary material for: Difference in Leukocyte Composition between Women before and after Menopausal Age, and Distinct Sexual Dimorphism
Source: PLoS One. 2016 Sep 22;11(9):e0162953. doi: 10.1371/journal.pone.0162953 (PMC5033487; doi:10.1371/journal.pone.0162953)
Supplement: S1 Table — (DOCX) [file pone.0162953.s001.docx]

**S1 Table. Total leukocyte counts in men and women in different age groups**

| Age group | Total leukocyte count (×10^9^ cells/L) | | *p*-value |
| --- | --- | --- | --- |
|  | Men | Women |  |
| ≤ 25 | 6.79 (1.59), n=3653 | 6.60 (1.64), n=3763 | 1.88×10^-8^ |
| 26-30 | 6.97 (1.68), n=3478 | 6.60 (1.66), n=2266 | 5.06×10^-19^ |
| 31-35 | 7.23 (1.81), n=2344 | 6.43 (1.64), n=1832 | 8.25×10^-53^ |
| 36-40 | 7.18 (1.79), n=3316 | 6.32 (1.57), n=2458 | 2.21×10^-84^ |
| 41-45 | 7.30 (1.88), n=3244 | 6.48 (1.56), n=2272 | 5.93×10^-66^ |
| 46-50 | 7.38 (2.01), n=2819 | 6.51 (1.68), n=2185 | 1.43×10^-64^ |
| 51-55 | 7.59 (2.00), n=2002 | 6.31 (1.78), n=1793 | 4.18×10^-109^ |
| 56-60 | 7.57 (2.05), n=1824 | 6.25 (1.54), n=1685 | 1.86×10^-103^ |
| 61-65 | 7.38 (2.08), n=1285 | 6.52 (1.77), n=1047 | 2.49×10^-28^ |
| 66-70 | 7.20 (1.79), n=824 | 6.45 (2.08), n=584 | 3.75×10^-17^ |
| ≥ 71 | 7.18 (1.98), n=1422 | 6.70 (1.74), n=780 | 4.10×10^-9^ |
| All subjects | 7.21 (1.87), n=26211 | 6.47 (1.66), n=20665 | <1.00×10^-300^ |

Data shown are mean (standard deviation) values.
