# Supplementary material for: Difference in Leukocyte Composition between Women before and after Menopausal Age, and Distinct Sexual Dimorphism
Source: PLoS One. 2016 Sep 22;11(9):e0162953. doi: 10.1371/journal.pone.0162953 (PMC5033487; doi:10.1371/journal.pone.0162953)
Supplement: S2 Table — (DOCX) [file pone.0162953.s002.docx]

**S2 Table. Monocyte counts and percentages in men and women in different age groups**

| Age group | Monocyte count (×10^9^ cells/L) | | *p*-value | Monocyte percentage | | *p*-value |
| --- | --- | --- | --- | --- | --- | --- |
|  | Men | Women |  | Men | Women |  |
| ≤ 25 | 0.47 (0.16), n=3652 | 0.42 (0.13), n=3764 | 2.46×10^-64^ | 7.00 (1.73), n=3652 | 6.43 (1.66), n=3764 | 6.43×10^-56^ |
| 26-30 | 0.49 (0.19), n=3479 | 0.41 (0.12), n=2265 | 7.36×10^-87^ | 7.01 (1.91), n=3479 | 6.31 (1.68), n=2266 | 2.47×10^-59^ |
| 31-35 | 0.49 (0.16), n=2344 | 0.40 (0.13), n=1832 | 7.06×10^-92^ | 6.87 (1.64), n=2344 | 6.32 (1.68), n=1832 | 7.60×10^-30^ |
| 36-40 | 0.49 (0.16), n=3315 | 0.40 (0.13), n=2458 | 1.60×10^-117^ | 6.88 (1.64), n=3316 | 6.44 (1.75), n=2458 | 1.80×10^-24^ |
| 41-45 | 0.49 (0.16), n=3243 | 0.40 (0.12), n=2272 | 2.53×10^-115^ | 6.81 (1.64), n=3244 | 6.31 (1.69), n=2273 | 3.61×10^-31^ |
| 46-50 | 0.51 (0.21), n=2818 | 0.40 (0.13), n=2185 | 2.82×10^-116^ | 6.92 (2.12), n=2819 | 6.28 (1.59), n=2185 | 2.43×10^-39^ |
| 51-55 | 0.52 (0.17), n=2002 | 0.38 (0.12), n=1792 | 7.52×10^-176^ | 6.90 (1.65), n=2002 | 6.07 (1.62), n=1792 | 3.18×10^-56^ |
| 56-60 | 0.51 (0.17), n=1823 | 0.37 (0.12), n=1685 | 4.20×10^-160^ | 6.83 (1.71), n=1824 | 6.05 (1.59), n=1685 | 2.87×10^-42^ |
| 61-65 | 0.50 (0.18), n=1285 | 0.39 (0.13), n=1047 | 1.16×10^-72^ | 6.90 (1.71), n=1285 | 6.05 (1.51), n=1047 | 6.11×10^-38^ |
| 66-70 | 0.50 (0.18), n=824 | 0.39 (0.13), n=584 | 1.67×10^-44^ | 7.06 (2.33), n=824 | 6.19 (1.62), n=584 | 6.28×10^-20^ |
| ≥ 71 | 0.52 (0.22), n=1421 | 0.43 (0.14), n=780 | 1.37×10^-33^ | 7.30 (2.01), n=1421 | 6.49 (1.79), n=780 | 4.70×10^-23^ |
| All subjects | 0.50 (0.18), n=26206 | 0.40 (0.13), n=20664 | <1.00×10^-300^ | 6.94 (1.80), n=26210 | 6.29 (1.67), n=20666 | <1.00×10^-300^ |

Data shown are mean (standard deviation) values.
