# Supplementary material for: Difference in Leukocyte Composition between Women before and after Menopausal Age, and Distinct Sexual Dimorphism
Source: PLoS One. 2016 Sep 22;11(9):e0162953. doi: 10.1371/journal.pone.0162953 (PMC5033487; doi:10.1371/journal.pone.0162953)
Supplement: S3 Table — (DOCX) [file pone.0162953.s003.docx]

**S3 Table. Eosinophil counts and percentages in men and women in different age groups**

| Age group | Eosinophil count (×10^9^ cells/L) | | *p*-value | Eosinophil percentage | | *p*-value |
| --- | --- | --- | --- | --- | --- | --- |
|  | Men | Women |  | Men | Women |  |
| ≤ 25 | 0.18 (0.15), n=3653 | 0.13 (0.13), n=3764 | 5.26×10^-70^ | 2.66 (2.02), n=3653 | 2.03 (1.72), n=3764 | 5.69×10^-64^ |
| 26-30 | 0.19 (0.15), n=3479 | 0.14 (0.13), n=2265 | 1.32×10^-62^ | 2.72 (1.97), n=3479 | 2.16 (1.75), n=2266 | 4.03×10^-46^ |
| 31-35 | 0.20 (0.18), n=2344 | 0.13 (0.12), n=1832 | 1.04×10^-80^ | 2.79 (2.11), n=2344 | 2.10 (1.76), n=1832 | 4.04×10^-49^ |
| 36-40 | 0.21 (0.16), n=3316 | 0.13 (0.11), n=2458 | 1.62×10^-138^ | 2.86 (2.00), n=3316 | 2.04 (1.67), n=2458 | 9.01×10^-88^ |
| 41-45 | 0.21 (0.17), n=3243 | 0.14 (0.11), n=2273 | 9.46×10^-101^ | 2.84 (2.02), n=3244 | 2.13 (1.70), n=2273 | 2.16×10^-61^ |
| 46-50 | 0.21 (0.20), n=2818 | 0.14 (0.12), n=2185 | 4.90×10^-94^ | 2.91 (2.37), n=2819 | 2.19 (1.81), n=2185 | 2.81×10^-52^ |
| 51-55 | 0.22 (0.16), n=2002 | 0.14 (0.11), n=1793 | 3.83×10^-80^ | 2.90 (1.99), n=2002 | 2.26 (1.69), n=1793 | 2.49×10^-31^ |
| 56-60 | 0.22 (0.24), n=1824 | 0.14 (0.11), n=1685 | 2.15×10^-71^ | 2.91 (2.36), n=1824 | 2.22 (1.68), n=1685 | 2.65×10^-27^ |
| 61-65 | 0.22 (0.18), n=1285 | 0.14 (0.12), n=1047 | 1.09×10^-36^ | 2.96 (2.24), n=1285 | 2.24 (1.87), n=1047 | 4.35×10^-21^ |
| 66-70 | 0.22 (0.21), n=824 | 0.14 (0.11), n=584 | 9.45×10^-24^ | 3.03 (2.55), n=824 | 2.23 (1.85), n=584 | 1.01×10^-13^ |
| ≥ 71 | 0.22 (0.23), n=1422 | 0.14 (0.11), n=780 | 2.12×10^-29^ | 3.05 (2.72), n=1422 | 2.20 (1.72), n=780 | 1.62×10^-22^ |
| All subjects | 0.20 (0.18), n=26210 | 0.14 (0.12), n=20666 | <1.00×10^-300^ | 2.84 (2.16), n=26212 | 2.14 (1.74), n=20667 | <1.00×10^-300^ |

Data shown are mean (standard deviation) values.
