# Supplementary material for: Difference in Leukocyte Composition between Women before and after Menopausal Age, and Distinct Sexual Dimorphism
Source: PLoS One. 2016 Sep 22;11(9):e0162953. doi: 10.1371/journal.pone.0162953 (PMC5033487; doi:10.1371/journal.pone.0162953)
Supplement: S4 Table — (DOCX) [file pone.0162953.s004.docx]

**S4 Table. Basophil Counts and percentages counts in men and women in different age groups**

| Age group | Basophil count (×10^9^ cells/L) | | *p*-value | Basophil percentage | | *p*-value |
| --- | --- | --- | --- | --- | --- | --- |
|  | Men | Women |  | Men | Women |  |
| ≤ 25 | 0.029 (0.020), n=3653 | 0.026 (0.017), n=3764 | 2.37×10^-13^ | 0.44 (0.30), n=3653 | 0.40 (0.26), n=3764 | 1.18×10^-7^ |
| 26-30 | 0.032 (0.035), n=3479 | 0.027 (0.017), n=2265 | 1.62×10^-17^ | 0.48 (0.54), n=3479 | 0.42 (0.27), n=2266 | 1.07×10^-5^ |
| 31-35 | 0.032 (0.023), n=2344 | 0.027 (0.017), n=1832 | 1.25×10^-17^ | 0.46 (0.33), n=2344 | 0.43 (0.27), n=1832 | 3.28×10^-2^ |
| 36-40 | 0.034 (0.024), n=3316 | 0.028 (0.018), n=2458 | 4.77×10^-31^ | 0.49 (0.42), n=3316 | 0.45 (0.30), n=2458 | 1.02×10^-4^ |
| 41-45 | 0.033 (0.022), n=3244 | 0.029 (0.023), n=2273 | 7.65×10^-13^ | 0.46 (0.31), n=3244 | 0.47 (0.40), n=2273 | 6.89×10^-1^ |
| 46-50 | 0.034 (0.034), n=2819 | 0.029 (0.027), n=2185 | 2.81×10^-19^ | 0.48 (0.63), n=2819 | 0.46 (0.45), n=2185 | 1.49×10^-2^ |
| 51-55 | 0.034 (0.029), n=2002 | 0.027 (0.019), n=1793 | 1.14×10^-24^ | 0.47 (0.40), n=2002 | 0.45 (0.38), n=1793 | 4.07×10^-1^ |
| 56-60 | 0.033 (0.024), n=1824 | 0.026 (0.016), n=1685 | 9.69×10^-27^ | 0.45 (0.30), n=1824 | 0.43 (0.25), n=1685 | 3.48×10^-1^ |
| 61-65 | 0.033 (0.039), n=1285 | 0.027 (0.016), n=1047 | 4.76×10^-8^ | 0.47 (0.64), n=1285 | 0.44 (0.26), n=1047 | 4.89×10^-1^ |
| 66-70 | 0.031 (0.020), n=824 | 0.029 (0.040), n=584 | 1.40×10^-5^ | 0.44 (0.27), n=824 | 0.49 (1.37), n=584 | 8.35×10^-1^ |
| ≥ 71 | 0.033 (0.021), n=1422 | 0.029 (0.018), n=780 | 1.20×10^-3^ | 0.47 (0.29), n=1422 | 0.45 (0.28), n=780 | 3.40×10^-1^ |
| All subjects | 0.033 (0.027), n=26212 | 0.027(0.020), n=20666 | 1.03×10^-162^ | 0.47 (0.43), n=26212 | 0.44 (0.39), n=20667 | 4.17×10^-16^ |

Data shown are mean (standard deviation) values.
