# Supplementary material for: Difference in Leukocyte Composition between Women before and after Menopausal Age, and Distinct Sexual Dimorphism
Source: PLoS One. 2016 Sep 22;11(9):e0162953. doi: 10.1371/journal.pone.0162953 (PMC5033487; doi:10.1371/journal.pone.0162953)
Supplement: S5 Table — (DOCX) [file pone.0162953.s005.docx]

**S5 Table. Neutrophil counts and percentages in men and women in different age groups**

| Age group | Neutrophil count (×10^9^ cells/L) | | *p*-value | Neutrophil percentage | | *p*-value |
| --- | --- | --- | --- | --- | --- | --- |
|  | Men | Women |  | Men | Women |  |
| ≤ 25 | 3.68 (1.21), n=3653 | 3.72 (1.35), n=3764 | 6.90×10^-1^ | 53.56 (8.16), n=3653 | 55.39 (8.66), n=3764 | 9.42×10^-21^ |
| 26-30 | 3.74 (1.25), n=3479 | 3.71 (1.38), n=2265 | 3.87×10^-2^ | 53.00 (7.87), n=3479 | 55.19 (8.59), n=2266 | 3.06×10^-22^ |
| 31-35 | 3.89 (1.35), n=2344 | 3.68 (1.31), n=1832 | 2.67×10^-9^ | 53.21 (7.92), n=2344 | 56.27 (8.22), n=1832 | 1.01×10^-33^ |
| 36-40 | 3.85 (1.30), n=3316 | 3.64 (1.22), n=2458 | 3.10×10^-11^ | 53.01 (7.64), n=3316 | 56.86 (7.92), n=2458 | 2.24×10^-75^ |
| 41-45 | 3.97 (1.37), n=3243 | 3.75 (1.24), n=2273 | 3.92×10^-10^ | 53.78 (7.62), n=3244 | 57.13 (7.76), n=2273 | 6.15×10^-56^ |
| 46-50 | 4.00 (1.50), n=2818 | 3.72 (1.30), n=2185 | 2.85×10^-14^ | 53.60 (8.13), n=2819 | 56.30 (8.08), n=2185 | 4.20×10^-31^ |
| 51-55 | 4.13 (1.44), n=2002 | 3.41 (1.15), n=1792 | 7.48×10^-71^ | 53.98 (8.16), n=2002 | 53.65 (8.36), n=1792 | 2.19×10^-1^ |
| 56-60 | 4.22 (1.55), n=1824 | 3.36 (1.14), n=1685 | 1.74×10^-81^ | 55.10 (8.14), n=1824 | 53.14 (8.30), n=1685 | 1.87×10^-12^ |
| 61-65 | 4.10 (1.59), n=1285 | 3.54 (1.40), n=1047 | 1.69×10^-23^ | 54.81 (8.43), n=1285 | 53.53 (8.65), n=1047 | 2.98×10^-4^ |
| 66-70 | 4.01 (1.35), n=824 | 3.54 (1.72), n=584 | 2.31×10^-14^ | 55.24 (8.34), n=824 | 54.05 (8.68), n=584 | 9.82×10^-3^ |
| ≥ 71 | 4.07 (1.53), n=1422 | 3.74 (1.26), n=780 | 3.20×10^-7^ | 56.02 (8.85), n=1422 | 55.46 (9.00), n=780 | 1.62×10^-1^ |
| All subjects | 3.92 (1.39), n=26210 | 3.64 (1.30), n=20665 | 2.61×10^-136^ | 53.80 (8.07), n=26212 | 55.45 (8.43), n=20666 | 1.68×10^-100^ |

Data shown are mean (standard deviation) values.
