# Supplementary material for: Difference in Leukocyte Composition between Women before and after Menopausal Age, and Distinct Sexual Dimorphism
Source: PLoS One. 2016 Sep 22;11(9):e0162953. doi: 10.1371/journal.pone.0162953 (PMC5033487; doi:10.1371/journal.pone.0162953)
Supplement: S6 Table — (DOCX) [file pone.0162953.s006.docx]

**S6 Table. Lymphocyte counts and percentages in men and women in different age groups**

| Age group | Lymphocyte count (×10^9^ cells/L) | | *p*-value | lymphocyte percentage | | *p*-value |
| --- | --- | --- | --- | --- | --- | --- |
|  | Men | Women |  | Men | Women |  |
| ≤ 25 | 2.43 (0.64), n=3653 | 2.31 (0.60), n=3764 | 5.58×10^-17^ | 36.35 (7.61), n=3653 | 35.77 (8.00), n=3764 | 1.29×10^-3^ |
| 26-30 | 2.53 (0.66), n=3479 | 2.31 (0.58), n=2265 | 1.57×10^-35^ | 36.82 (7.41), n=3479 | 35.95 (8.06), n=2266 | 3.79×10^-5^ |
| 31-35 | 2.61 (0.70), n=2344 | 2.19 (0.58), n=1832 | 1.11×10^-88^ | 36.69 (7.52), n=2344 | 34.89 (7.71), n=1832 | 3.88×10^-14^ |
| 36-40 | 2.60 (0.70), n=3316 | 2.12 (0.57), n=2458 | 5.51×10^-171^ | 36.78 (7.14), n=3316 | 34.21 (7.31), n=2458 | 2.76×10^-40^ |
| 41-45 | 2.59 (0.70), n=3243 | 2.16 (0.56), n=2273 | 2.28×10^-129^ | 36.13 (7.13), n=3244 | 33.98 (7.12), n=2273 | 5.84×10^-28^ |
| 46-50 | 2.62 (0.76), n=2818 | 2.22 (0.61), n=2185 | 5.59×10^-88^ | 36.11 (7.58), n=2819 | 34.80 (7.61), n=2185 | 1.59×10^-9^ |
| 51-55 | 2.68 (0.84), n=2002 | 2.33 (0.65), n=1792 | 8.55×10^-43^ | 35.78 (7.74), n=2002 | 37.59 (7.84), n=1792 | 1.02×10^-12^ |
| 56-60 | 2.59 (0.77), n=1824 | 2.35 (0.64), n=1685 | 3.06×10^-19^ | 34.74 (7.63), n=1824 | 38.19 (7.80), n=1685 | 4.37×10^-39^ |
| 61-65 | 2.52 (0.77), n=1285 | 2.42 (0.68), n=1047 | 3.77×10^-3^ | 34.89 (8.02), n=1285 | 37.78 (8.17), n=1047 | 1.76×10^-17^ |
| 66-70 | 2.43 (0.82), n=824 | 2.35 (0.77), n=584 | 3.45×10^-2^ | 34.26 (8.10), n=824 | 37.04 (8.18), n=584 | 3.18×10^-10^ |
| ≥ 71 | 2.34 (0.78), n=1422 | 2.36 (0.84), n=780 | 9.47×10^-1^ | 33.19 (8.52), n=1422 | 35.42 (8.71), n=780 | 5.58×10^-9^ |
| All subjects | 2.55 (0.73), n=26210 | 2.26 (0.62), n=20665 | <1.00×10^-300^ | 35.98 (7.62), n=26212 | 35.71 (7.89), n=20666 | 1.59×10^-4^ |

Data shown are mean (standard deviation) values.
