# Supplementary material for: Difference in Leukocyte Composition between Women before and after Menopausal Age, and Distinct Sexual Dimorphism
Source: PLoS One. 2016 Sep 22;11(9):e0162953. doi: 10.1371/journal.pone.0162953 (PMC5033487; doi:10.1371/journal.pone.0162953)
Supplement: S7 Table — (DOCX) [file pone.0162953.s007.docx]

**S7 Table. Neutrophil-to-lymphocyte ratio in men and women in different age groups**

| Age group | Neutrophil/lymphocyte ratio | | *p*-value |
| --- | --- | --- | --- |
|  | Men | Women |  |
| ≤ 25 | 1.61 (0.70), n=3653 | 1.70 (0.82), n=3764 | 3.73×10^-9^ |
| 26-30 | 1.56 (0.67), n=3479 | 1.70 (0.80), n=2265 | 3.43×10^-11^ |
| 31-35 | 1.58 (0.73), n=2344 | 1.78 (0.94), n=1832 | 2.83×10^-21^ |
| 36-40 | 1.55 (0.63), n=3316 | 1.82 (0.92), n=2458 | 1.30×10^-53^ |
| 41-45 | 1.60 (0.61), n=3243 | 1.82 (0.74), n=2273 | 2.75×10^-38^ |
| 46-50 | 1.62 (0.77), n=2818 | 1.76 (0.71), n=2185 | 1.47×10^-16^ |
| 51-55 | 1.65 (0.83), n=2002 | 1.57 (0.84), n=1792 | 5.31×10^-6^ |
| 56-60 | 1.73 (0.73), n=1824 | 1.52 (0.72), n=1685 | 5.00×10^-26^ |
| 61-65 | 1.75 (0.90), n=1285 | 1.56 (0.75), n=1047 | 7.79×10^-11^ |
| 66-70 | 1.80 (1.07), n=824 | 1.61 (0.80), n=584 | 1.59×10^-6^ |
| ≥71 | 1.91 (0.97), n=1422 | 1.76 (0.92), n=780 | 7.09×10^-5^ |
| All subjects | 1.63 (0.75), n=26210 | 1.71 (0.82), n=20665 | 2.34×10^-28^ |

Data shown are mean (standard deviation) values.
